# Effects of Radiation Therapy and Comorbidity on Health-Related Quality of Life and Mortality Among Older Women With Low-Risk Breast Cancer: Protocol for a Retrospective Cohort Study

**DOI:** 10.2196/18056

**Published:** 2020-11-12

**Authors:** Eunkyung Lee, Robert B Hines, Jean L Wright, Eunji Nam, Michael J Rovito, Xinliang Liu

**Affiliations:** 1 College of Health Professions and Sciences University of Central Florida Orlando, FL United States; 2 College of Medicine University of Central Florida Orlando, FL United States; 3 Department of Radiation Oncology and Molecular Radiation Sciences Johns Hopkins University Baltimore, MD United States; 4 Department of Social Welfare Incheon National University Incheon Republic of Korea; 5 College of Community Innovation and Education University of Central Florida Orlando, FL United States

**Keywords:** radiation therapy, comorbidity, health-related quality of life, survival, early-stage breast cancer, older women

## Abstract

**Background:**

The National Comprehensive Cancer Network Breast Cancer Guidelines Committee suggests that the omission of adjuvant radiation therapy (RT) after breast-conserving surgery can be a reasonable option among older women with low-risk breast cancer (early-stage, estrogen receptor-positive, and node-negative) if they are treated with endocrine therapy. However, RT usage in this group of women still exceeds 50%. Conversely, older women tend to forego RT (even when necessary) due to cost, inconvenience, and potential adverse responses associated with RT. Understanding health-related quality of life (HRQOL) change with receipt of RT among older women in the modern era is limited due to the under-representation of this population in clinical trials.

**Objective:**

The proposed study aims to examine the associations of RT with HRQOL trajectories as well as survival outcomes among older women with 5-10 years of follow-up. We will also assess whether prediagnosis comorbidity burden influences receipt of RT and whether the associations between RT and HRQOL trajectory and survival outcomes are modified by the comorbidity burden.

**Methods:**

We will use a retrospective cohort study design with the population-based Surveillance, Epidemiology, and End-Results database linked to the Medicare Health Outcomes Survey (SEER-MHOS). Older women (≥65 years) who were diagnosed with low-risk breast cancer in 1998-2014, received breast-conserving surgery, and participated in MHOS 1998-2016 are eligible for this analysis. The latent class analysis clustering method will be used to identify each patient’s prediagnosis comorbidity burden, and HRQOL will be evaluated using the Short Form 36/Veterans RAND 12-Item Health Survey scales. The inverse-weighted estimates of the probability of treatment will be included to control for treatment selection bias and confounding effects in subsequent analysis. The association of RT with HRQOL trajectory will be evaluated using inverse-weighted multilevel growth mixture models. The inverse-weighted Cox regression model will be used to obtain hazard ratios with 95% CIs for the association of RT with survival outcomes. Differential effects of RT on both outcomes according to comorbidity burden class will also be evaluated.

**Results:**

As of October 2020, the study was approved by the institutional review board, and SEER-MHOS data were obtained from the National Cancer Institute. Women with low-risk breast cancer who met inclusion and exclusion criteria have been identified, and prediagnosis comorbidity burden class has been characterized using latent class analysis. Further data analysis will begin in November 2020, and the first manuscript will be submitted in a peer-reviewed journal in February 2021.

**Conclusions:**

This research can potentially improve clinical outcomes of older women with low-risk breast cancer by providing them additional information on the HRQOL trajectories when they make RT treatment decisions. It will facilitate informed, shared treatment decision making and cancer care planning to ultimately improve the HRQOL of older women with breast cancer.

**International Registered Report Identifier (IRRID):**

DERR1-10.2196/18056

## Introduction

Breast cancer is the most prevalent cancer and the second leading cause of cancer-related deaths in American women [1]. Due to the aging of the female population and the increasing incidence of breast cancer with age [2], the number of older women with breast cancer continues to grow. Older women diagnosed with early-stage breast cancer have multiple locoregional treatment options depending on prognostic factors such as tumor type and disease stage. Adjuvant radiation therapy (RT) following breast-conserving surgery (BCS) is standard treatment for early-stage breast cancer, as this course of treatment improves clinical outcomes significantly [3,4]. Older women are often concerned with potential health-related quality of life (HRQOL) declines associated with cancer treatment [5]. These concerns of older women can present clinical challenges when making treatment decisions considering the pros and cons of adjuvant RT within the context of life expectancy.

Many randomized controlled trials (RCTs) have focused on the cancer care of older women. One of them is the CALGB 9343 trial, which reported that the recurrence rates of older women with estrogen receptor (ER)-positive early-stage breast cancer who received endocrine therapy alone were not significantly different from those of women who received both endocrine therapy and RT (4% vs 1%) [6]. Moreover, no significant survival benefits were observed with the addition of RT to endocrine therapy compared to endocrine therapy alone [6]. These remarkable results from the CALGB 9343 trial led to a change in the National Comprehensive Cancer Network (NCCN) Breast Cancer Guidelines in 2004, which suggested the omission of RT as a reasonable option for older women with low-risk breast cancer (early-stage, ER-positive, and node-negative) if they are treated with endocrine therapy [7]. Additionally, 10-20 years of long-term follow-up results of early RCT studies, such as CALGB9343, PRIME II, and BASO II, have further confirmed that adjuvant RT can be omitted in older women with favorable early-stage ER-positive, node-negative breast cancer [8-10].

Despite the aforementioned guideline update, the treatments of older women with low-risk breast cancer vary widely. In prior studies, the patterns of RT omission were not consistent across institutions, even at NCCN member institutions [11-13]. More importantly, RT usage in this group of women still exceeds 50% [14]. A recent survey reported that high proportions of surgeons and radiation oncologists erroneously overestimated survival benefits associated with RT in this group of women [15] because the estimation of life expectancy in older women is not straightforward [11]. Many treatment-decision algorithms include age to estimate life expectancy, but chronological age is not always correlated with one’s biological age and mortality. Including geriatric assessment in the algorithm improved compliance with NCCN guidelines without diminishing survival benefits [16]. Since accompanying comorbid conditions at diagnosis are strongly associated with mortality regardless of age [17-19], considering comorbidity burden in addition to age and tumor characteristics will improve the selection of women who can omit RT.

On the other hand, some older women tend to forego RT (even when necessary) due to cost, inconvenience, and uncertainty of potential adverse responses associated with RT. Limited evidence suggests that older women are more concerned about HRQOL declines rather than fear of recurrence when they make treatment decisions [20,21]. However, understanding HRQOL change with the addition of RT among this population is limited due to the under-representation of older women and those with comorbidities in clinical trials [22,23]. Many studies have evaluated adverse effects on HRQOL with receipt of RT, but most of them were cross-sectional designs or assessments of short-term changes within 1 year post RT [18,24-28]. Not many studies have evaluated the long-term effects of RT on HRQOL. In an older study, Lundstedt and colleagues reported that 10.3% of women had weekly pain even after 10-17 years of RT, and this observation was significantly higher among women who received adjuvant RT than those who did not (1.7%, *P*=.001) [29]. Therefore, we need to update long-term HRQOL with more data since there have been many advancements in RT techniques over the past 3 decades. Moreover, baseline HRQOL is a major predictor of subsequent HRQOL; thus, longitudinal evaluation of HRQOL trajectory in a cohort study design, rather than a cross-sectional evaluation of post-treatment HRQOL, will provide more valid estimates of the impact of RT on HRQOL.

Few studies have investigated the effect of RT among older women with varying comorbidity burden, and to our knowledge, none of the previous studies have evaluated long-term HRQOL trajectories post RT within older women treated in the modern era. Thus, the proposed study aims to examine the effects of RT on long-term HRQOL trajectories and survival outcomes among older women with low-risk breast cancer in an observational setting. Minimizing the potential bias due to treatment selection and controlling for potential differences in confounding factors between treatment groups are essential in observational comparative effectiveness studies without randomized treatment [30]. To mitigate this concern, first, we will only select older women with low-risk breast cancer whose main treatment plan includes BCS and endocrine therapy with RT being an option. Among this group of women, only 3.3% undergo chemotherapy [1]; thus, the confounding effect of chemotherapy on HRQOL and survival outcomes is minimal. Second, to further balance out the potential confounding factors between the 2 groups, we will use inverse probability of treatment weighting (IPTW) methods, a type of propensity score analysis [31-34], which we have applied successfully in other projects [35,36]. All potential confounding variables will be included in the statistical models when deriving the weights for each group.

This comparative effectiveness study will utilize a national sample of older women to provide much-needed evidence regarding the combined effects of RT and comorbidity on HRQOL (subjective outcome) and survival (objective outcome) in older women. This research has 2 aims.

Regarding Aim 1, we will examine the effect of RT on the HRQOL trajectory over time and its interaction with comorbidity burden in older women with low-risk breast cancer (n=465) using IPTW-adjusted growth mixture models. We hypothesize that the HRQOL trajectory will differ by treatment status and comorbidity burden, and the largest decline will be observed in older women with the highest comorbidity burden.

Regarding Aim 2, we will examine the effect of RT on 5-year mortality and its interaction with comorbidity burden in older women with low-risk breast cancer (n=634) using Kaplan-Meier survival analysis and a IPTW-adjusted Cox regression model. We hypothesize that receiving adjuvant RT will improve 5-year mortality, but the magnitude of improvement will differ by comorbidity burden class. We expect that women who have the lowest comorbidity burden and also receive RT will experience the greatest improvement in survival outcomes.

## Methods

### Overview

**Figure 1:**
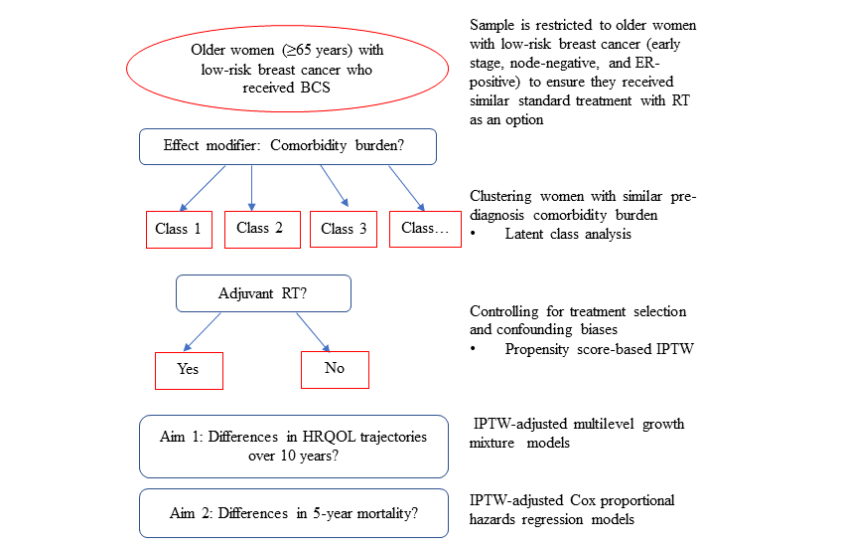
Overview of the proposed retrospective cohort study design. The study population will comprise older women with low-risk breast cancer who received breast-conserving surgery (BCS) as a primary treatment in 1998-2014 and took part in Medicare Health Outcomes Survey (MHOS). The women were first clustered according to their prediagnosis comorbidity burden using latent class analysis. Next, the propensity score-based probability of receiving adjuvant radiotherapy (RT) will be obtained, and inverse probability of treatment weights (IPTW) will be estimated and included in statistical models. To examine the effects of RT on 2 outcomes, health-related quality of life (HRQOL) trajectories over 10-year follow-up periods and 5-year survival outcomes, IPTW-adjusted multilevel growth mixture models and IPTW-adjusted Cox proportional hazards regression models will be used, respectively. The data are sourced from the SEER-MHOS Database. ER: estrogen receptor.

This observational study uses a retrospective cohort design, and the study population comprises older women with low-risk breast cancer who received BCS as a primary treatment and took part in the Medicare Health Outcomes Survey (MHOS). As depicted in Figure 1, the women are first clustered according to their prediagnosis comorbidity burden. Next, we will obtain the propensity score-based probability of receiving adjuvant RT based on observed potential confounding variables. Then, inverse probability weights will be calculated and adjusted for in subsequent analyses. We will examine the effect of RT on 2 outcomes: differences in the patterns of HRQOL trajectories over 10 years post diagnosis and 5-year survival outcomes.

We will utilize the Surveillance, Epidemiology, and End-Results (SEER) database linked to the Medicare Health Outcomes Survey (MHOS) for this project. The SEER-MHOS database resulted from the collaborative efforts of the National Cancer Institute (NCI) and the Centers for Medicare & Medicaid Services. SEER collects data on cancer incidence, treatment, and survival, covering nearly 28% of the United States population. SEER includes high-quality, reliable patient characteristic data (eg, demographics, initial treatment and treatment sequence, tumor characteristics, vital status, and cause of death), while the MHOS data provide patient-reported HRQOL outcomes, comorbidity, functional status, and symptoms for a randomly selected sample of the Medicare Advantage Organization enrollees. MHOS is a longitudinal, 2-wave survey administered twice within a cohort at a 2-year interval. Participants who responded to the first baseline survey may have completed the second follow-up survey. In addition, some participants were sampled in more than 1 cohort, resulting in multiple surveys in different years, which allows the evaluation of HRQOL change over time. Therefore, SEER-MHOS is the best available data source for examining the long-term HRQOL trajectory after diagnosis of cancer in a diverse elderly population with varying degrees of comorbidity burden even though SEER-MHOS is limited in its geographical coverage of the United States.

This study has been approved by the Institutional Review Board of the University of Central Florida (approval number: SBE-18-14238). As individuals who participated in MHOS have provided informed consent to participate in research, this study is exempt from the additional requirements of obtaining informed consent from the study participants [37].

### Aim 1: Examine the Effect of RT on HRQOL Trajectory Over Time

#### Study Design and Study Population

A retrospective cohort study design is planned for Aim 1. Older women who were enrolled in Medicare and completed the MHOS between 1998 and 2016 are eligible for the study. The inclusion criteria were as follows: (1) a diagnosis of primary breast cancer between 1998 and 2014 and reported to SEER, (2) low-risk breast cancer, including early-stage (T1-2N0), ER-positive, and node-negative, (3) ≥65 years of age at diagnosis (with 65 years being the threshold for Medicare eligibility), (4) receipt of BCS (lumpectomy, quadrantectomy, or partial mastectomy) as primary treatment for breast cancer, and (5) completion of at least 1 MHOS assessment before and after breast cancer diagnosis to allow for assessing the change in HRQOL. Cases were excluded if they (1) had a previous cancer diagnosis (other than nonmelanoma skin cancer), (2) had more than 1 primary tumor at diagnosis, (3) received treatment outside of standard time frames (ie, treatment initiated 1 year after cancer diagnosis), (4) received RT before surgery, or (5) tumor histology was other than carcinoma. While these eligibility criteria may reduce external validity, they will enhance internal validity.

#### Measurements

The primary exposure variable will be adjuvant RT. We will consider BCS alone and BCS with adjuvant RT as the treatments. The SEER data provide information on radiation treatment and the sequence of surgery and radiation.

The outcomes of Aim 1 will be HRQOL scores measured over a 5-10 years of follow-up (ie, prediagnosis through post-treatment). MHOS measures HRQOL using the Medical Outcomes Study 36-item Short Form (SF-36) through the year 2005 and the Veterans RAND 12-item Health Survey (VR-12) from the year 2006 onwards. These 2 questionnaires have been validated in many languages and are widely used in HRQOL research, including that involving breast cancer patients [38]. The NCI developed algorithms to convert SF-36 scales to VR-12 scales; hence, the latter will be used across all cohorts in the current study. The VR-12 items correspond to 8 health domains, namely physical functioning, role limitations due to physical problems, role limitations due to emotional problems, bodily pain, social functioning, mental health, vitality, and perceptions of general health. These 12 items are then summarized into 2 summary scores: the physical component summary score and the mental component summary score. These 2 summary scores are standardized scores (mean 50, SD 10), which are normed based on the general US population. A total of 465 women who met the eligibility criteria for Aim 1 completed MHOS multiple times (an average of 3 times). We will sort the data according to the prediagnosis (T_0_) and postdiagnosis (T_1_…T_10_) timelines, specifying the time lapse from the cancer diagnosis. The prediagnosis HRQOL (T_0_, n=465) will be assessed using the survey conducted within 24 months prior to the diagnosis date recorded in SEER. The postdiagnosis HRQOL (T_1_…T_10_) will be assessed using the survey completed after the diagnosis of breast cancer. We collected 465 data points for the precancer diagnosis, 508 data points within 2 years postdiagnosis, 166 data points for 2-<5 years postdiagnosis, and 92 data points for ≥5 years postdiagnosis. The number of patients who completed MHOS in each time frame is presented in Table 1.

**Table 1:** Number of patients who completed MHOS in each time frame (N=465).

Survey time to cancer diagnosis (months), t	Prediagnosis and postdiagnosis HRQOL timelines	n (%)
–24<t<0	T_0_	465 (100)
0≤*t*<12	T_1_	253 (54)
12≤*t*<24	T_2_	255 (55)
24≤*t*<36	T_3_	79 (17)
36≤*t*<48	T_4_	50 (11)
48≤*t*<60	T_5_	37 (8)
60≤*t*<72	T_6_	29 (6)
72≤*t*<84	T_7_	21 (5)
84≤*t*<96	T_8_	17 (4)
96≤*t*<108	T_9_	8 (2)
108≤t	T_10_	17 (4)

We considered comorbidity burden as the effect modifier. Prediagnosis comorbidity is an important component of this study, as it likely impacts HRQOL and survival outcomes adversely while also affecting treatment decisions. The presence and number of comorbidities as well as their severity are important. However, the severity of each comorbidity is not available from MHOS, whereas the functional limitations and symptoms related to these comorbidities are. To account for functional limitations and symptoms as a component of comorbidity, we created a new variable named “comorbidity burden class” with 24 self-reported items (ie, 12 chronic medical conditions, 7 functional difficulties, and 5 symptoms) in MHOS (Textbox 1) using latent class analysis. This technique has been applied successfully to identify healthy patients or comorbidity profiles of cancer patients in studies using SEER-MHOS and the National Cancer Database [39,40] when the severity of comorbid conditions is not available. To identify the prediagnosis comorbidity burden class, the survey completed within 24 months before cancer diagnosis was evaluated. Latent class analysis is a subset of structural equation modeling, which is used to categorize subjects into a set of homogeneous groups (eg, k distinct membership classes of comorbidity) according to the observed variables (eg, self-reported chronic medical conditions, functional status, and symptoms). We anticipated that this procedure would suggest 3-5 distinct categories. We selected the most parsimonious number of classes using the Bayesian information criterion to find the best fitting numbers of classes. The classes of comorbidity burden will be tested as effect modifiers.

**Items included in latent class analysis modeling for comorbidity burden class.**

**Functional status**

- Difficulty in moderate activities
- Difficulty bathing
- Difficulty dressing
- Difficulty eating
- Difficulty using the toilet
- Difficulty walking
- Difficulty getting in/out of chairs
- Chest pain with exertion
- Chest pain at rest
- Short of breath at rest
- Short of breath walking
- Short of breath with stairs

**Chronic medical conditions**

- Angina pectoris/coronary artery disease
- Hypertension
- Myocardial infarction
- Congestive heart failure
- Stroke
- Emphysema, asthma, or chronic obstructive pulmonary disease
- Diabetes, high blood sugar, or sugar in urine
- Crohn disease, ulcerative colitis, or inflammatory bowel disease
- Arthritis of hip/knee
- Heart attack
- Sciatica
- Depression

Based on a literature review, potential confounders included age, race/ethnicity, smoking, body mass index, socioeconomic status (income and education), SEER region, rurality, lag time since treatment, disease stage, tumor grade, and molecular subtype. The year of diagnosis was also included as a covariate in the statistical model to control for variability in diagnostic or treatment criteria over 17 years (1998-2014). Demographics, general information pertaining to treatment status (surgery and radiation), and tumor-related factors (stage, grade, and tumor molecular subtype) were obtained from sources such as the SEER Patient Entitlement and Diagnosis Summary File, the Enrollment Database maintained by the Centers for Medicare & Medicaid Services, and self-reported information.

#### Statistical Analysis Plan

Regarding the descriptive analyses, the baseline characteristics of the study population will be described in terms of the mean and SD for continuous variables and frequencies with percentages for categorical variables. Descriptive analyses will be conducted to compare demographic, tumor-related, and treatment-related factors for the study population by their treatment status (BCS and RT vs BCS only). Differences in continuous variables and categorical variables will be assessed by *t* tests and chi-square tests, respectively. All analyses will be conducted using SAS 9.4 (SAS Institute Inc.) and R 4.0.1 (R Core Team), and a 2-tailed α level of .05 will denote statistical significance.

The probability of the treatment received by a particular person will be estimated by generalized boosted models using receipt of RT as the dependent variable and the baseline characteristics, such as age at diagnosis, race/ethnicity, smoking status, income, education, SEER region, rurality, disease stage, tumor grade, tumor subtype, and year of diagnosis, as the independent variables. Then, the inverse of the probability of receiving the treatment the patient actually received conditional on the observed covariates will be calculated and included as a weight for each patient in subsequent analyses [41]. Balances in potential confounding factors between the treatment groups will be assessed using diagnostic tools, including chi-square tests for categorical variables, *t* tests for continuous variables, and the standardized mean differences between 2 groups [42]. The standardized mean difference will be calculated as the difference in means or proportions divided by a pooled estimate of SD. A standardized mean difference≥10% will be considered as evidence of imbalance [43].

Regarding the estimation of RT and comorbidity effects on HRQOL trajectory, we will use a multilevel growth mixture model with surveys clustered within the women to test whether a difference exists in the patterns of HRQOL trajectories with receipt of RT and comorbidity class after controlling for confounders. At level 1, we will first regress woman i’s HRQOL change, which describes how each woman’s HRQOL depends on time t. This procedure will provide the intercept and slope of the time predictor for each woman. At level 2, we will regress each woman’s intercept and slope on her RT treatment (yes/no) and comorbidity class, adjusted for confounders. This procedure will feature how individual HRQOL change trajectories vary across treatment groups (RT) and comorbidity burden classes (COM). Equation (1) presents a simplified model with no covariates, predicting HRQOL from years after diagnosis (TIME), radiation treatment (RT), and comorbidity class (COM). It will estimate the intercepts and slopes for the group of women.

HRQOL_ti_ = γ_00_ + γ_01_RT_it_ + γ_02_COM_i_ + γ_10_TIME_it_ + γ_11_RT_i_TIME_it_ + γ_12_COM_i_TIME_it_ + error **(1)**

where

γ_00_=Estimated average HRQOL in the year of diagnosis for women who did not receive RT

γ_01_=Value added to the average HRQOL if the women received RT

γ_02_=Value added to the average HRQOL due to the comorbidity class

γ_10_=Change in HRQOL for all women for each year from diagnosis

γ_11_=Additional change for each year if the women received RT

γ_12_=Additional change for each year due to the comorbidity class

A significant negative value on γ_11_ will indicate that women who receive RT experience steeper declines in HRQOL over time than those who did not receive RT, while a significant negative value on γ_12_ will indicate stronger HRQOL declines due to the comorbidity burden.

We will also test for the existence of a differential effect of RT on the HRQOL trajectory according to the comorbidity burden class by incorporating interaction terms in the model. If we lack the power to detect statistically significant differences according to comorbidity level but point estimates for the effect of RT indicate that meaningful differences are present across levels of comorbidity, separate estimates will be reported for each comorbidity class. As each participant completed MHOS at different time points, growth mixture modeling is more flexible and sensitive than traditional repeated-measures analysis [44]. It can also capture nonlinear patterns [44].

Regarding the sensitivity/subgroup analysis, if the sample size allows, subgroup analysis by age and tumor stage will be conducted to examine whether the findings are generalizable to all patients satisfying the eligibility criteria.

### Aim 2: Examine the Effect of RT on Breast Cancer-Specific Mortality

#### Study Design and Study Population

A retrospective cohort study design will be used in the context of Aim 2. Older women who were enrolled in Medicare and completed the MHOS will be selected for this study. The inclusion criteria were (1) a diagnosis of primary breast cancer between 1998 and 2010 (to ensure at least 5 years of follow-up after a cancer diagnosis) and report to SEER, (2) a diagnosis of low-risk breast cancer, (3) ≥65 years of age at diagnosis given the Medicare framework, (4) receipt of BCS (lumpectomy, segmental resection, quadrantectomy, or partial mastectomy) as primary treatment for breast cancer, and (5) completion of at least 1 MHOS within 24 months prior to breast cancer diagnosis (to allow the evaluation of the prediagnosis comorbidity burden). Participants will be excluded from the study if (1) they had a previous cancer diagnosis (other than nonmelanoma skin cancer), (2) they had more than 1 primary tumor at diagnosis, (3) they received treatment outside of standard time frames (ie, treatment initiated 1 year after cancer diagnosis), (4) they received RT before surgery, or (5) their vital status was missing.

#### Measurements

The exposure variables are the same as those of Aim 1, namely BCS alone and BCS with adjuvant RT. We will consider vital status and cause of death as the outcomes. The main outcome for Aim 2 will be mortality (all-cause mortality and breast cancer-specific mortality considering nonbreast cancer mortality as a competing event). The SEER data file provides the vital status of participants and the date and causes of death, which have been verified through the National Death Index. Survival will be calculated from the date of diagnosis to the last known follow-up date or December 31, 2016, whichever comes first. As in the case of Aim 1, the comorbidity burden will serve as the effect modifier. The confounding factors will match those of Aim 1.

#### Statistical Analysis Plan

The descriptive analyses outlined for Aim 1 will apply to Aim 2 as well. Regarding the IPTW, we will apply the same procedures as in Aim 1 to the dataset to obtain a weight for each woman. The effect of RT on mortality will be assessed via 5-year survival curves of the study population using the Kaplan-Meier method, which will incorporate the IPTW. To estimate the effect of RT and comorbidity on mortality, we will use the weighted Cox proportional hazards model to obtain the IPTW-adjusted relative hazard of 5-year overall and cause-specific mortality from the date of diagnosis. IPTW-adjusted subdistribution hazard ratios with 95% CIs will be reported. The proportional hazards assumption will be assessed using the Schoenfeld residuals test, which measures the correlation between residuals of each covariate and time and evaluates the interactions with time in the survival models. Sensitivity analysis will be performed to examine whether the censoring may be informative rather than noninformative. We will consider 2 extreme possibilities: events occurred immediately after censoring or later than any other observed events.

### Limitations

A significant limitation of this study concerns the lack of data on chemotherapy, endocrine therapy, or other systemic therapy information from the SEER data, which may have a major impact on HRQOL and survival outcomes. To minimize the confounding effects from other treatments on HRQOL, we restricted our study population to women with low-risk, favorable breast cancer whose treatment plan would typically not involve chemotherapy. We assumed that most women in the study population received endocrine therapy according to their ER-positive status. Another limitation concerns the SEER-MHOS data; although they are population-based data, SEER covers certain geographic areas, and MHOS only samples insured women; thus, the overlapping of the 2 databases could be very limited, and only some women/tumor characteristics will be considered in the analysis. However, to the best of our knowledge, this is the best resource to evaluate the long-term effect on HRQOL from RT at the population level.

## Results

As of October 2020, the study was approved by the Institutional Review Board of the University of Central Florida (SBE-18-14238), and the permission to use the SEER-MHOS database was obtained from NCI and SEER. Data have been curated for data analysis, women with low-risk breast cancer who met the inclusion and exclusion criteria have been identified, and prediagnosis comorbidity burden class has been characterized by latent class analysis. Further data analysis will begin in November 2020. The results of this study will be disseminated in peer-reviewed journals and presented at medical/scientific conferences. The first set of results of this study, namely those concerning prediagnosis comorbidity burden and HRQOL, will be submitted for publication in peer-reviewed journals in February 2021, and the second set of results regarding the effect of RT on survival outcomes will be submitted in June 2021.

## Discussion

This study will provide empirical evidence for the effect of RT on HRQOL trajectory as well as survival outcomes using comparative effectiveness research methods with patient-reported HRQOL outcomes, which provide patients’ perspectives on health care options. This research has the potential to improve clinical outcomes of older women with cancer enrolled in Medicare Advantage Organizations by providing information on HRQOL trajectories in addition to potential survival advantages from RT. This project will contribute new knowledge of long-term HRQOL to the existing evidence concerning the use of adjuvant RT after BCS in older women with low-risk breast cancer. By examining the independent and potential interactive effects of RT and comorbidity on HRQOL trajectories and survival outcomes, this evidence can facilitate communication between older women and their health care providers for shared decision making.

### Data Statement

The data analyzed in this study are available in the NCI’s repository [45]. Permission to use the data is required by the NCI. Investigators interested in using SEER-MHOS data must submit a research protocol as part of their data request.

## Abbreviations

BCS: breast-conserving surgery
ER: estrogen receptor
HRQOL: health-related quality of life
IPTW: inverse probability of treatment weighting
MHOS: Medicare Health Outcomes Survey
NCCN: National Comprehensive Cancer Network
NCI: National Cancer Institute
RCT: randomized controlled trial
RT: radiation therapy
SEER: Surveillance, Epidemiology, and End Results
